# 

**Published:** 1891-09

**Authors:** 


### CONTENTS:

Article I.—A Plea for Conservatism. By W. C. Barrett, M. D. D. D. S. - - - - - - - 193
" II.—Physiological Action of Obtundents. By L. E. Custer, D. D. S. - - - - - - 203
" III.—Sterilization of Dental Instruments. - - - 209
" IV.—Diseases of the Oral Mucous Membrane. By. J. D. Patterson, D. D. S. - - - - - 212
" V.—Notes on the Anatomy, Physiology and Pathological Significance of Pain and other Nervous Phenomena In Relation to Dental Disease. By Thomas Gaddes, M. D., L. D. S. - - - - - 219
" VI.—Disease of the Antrum. By S. Woolverton, L. D. S. 227
" VII.—Notes on a Case of Alveolar Abscess, Complicated with Repeated and Profuse Hæmorrhage and Fracture of Inferior Maxilla. By Herbert R. Bowtell, L. D. S. - - - - - - - 231

### COMMUNICATIONS.

American Medical Association. - - - - - - 236

### OBBITUARY.

Samuel Cartwright, F. R. C. S. - - - - - - 236

### MONTHLY SUMMARY.

Covering Gold Crowns with Glass. - - - - - 238
A Gold Clasp, Perfect in Fit, Easily Made, and not Wearing to the Tooth Clasped. - - - - - - - - - 239
Observations on the Decay of the Teeth of Young Mothers. - 239
The United States Supreme Court Decision in "Tooth Crowns." - 240

